# Worse prognosis in young patients with locally advanced rectal cancer following neoadjuvant chemoradiotherapy: A comparative study

**DOI:** 10.1097/MD.0000000000021304

**Published:** 2020-08-28

**Authors:** Yiyi Zhang, Ye Wang, Xing Liu, Bin Chen, Jinfu Zhuang, Shoufeng Li, Yuanfeng Yang, Yibin Su, Guoxian Guan

**Affiliations:** a Department of Colorectal Surgery, The First Affiliated Hospital of Fujian Medical University; b Department of Colorectal Surgery, Fujian Medical University Union Hospital, Fuzhou; c Department of Gastrointestinal Surgery, Quanzhou First Hospital Affiliated to Fujian Medical University, Quanzhou, China.

**Keywords:** age, neoadjuvant chemoradiotherapy, prognosis, rectal cancer

## Abstract

To determine the efficacy of neoadjuvant chemoradiotherapy (NCRT) between young and old patients with locally advanced rectal cancer (LARC) in terms of tumor response and survival outcome.

LARC patients undergoing NCRT and radical surgery from 2011 to 2015 were included and divided into: young (aged ≤50 years) and old group (aged >50 years). Multivariate analyses were performed to identify risk factors for local recurrence. Least absolute shrinkage and selection operator analysis was performed to identify risk factors for overall survival. Predicting nomograms and time-indepent receiver operating characteristic curve analysis were performed to compare the models containing with/withour age groups.

A total of 572 LARC patients were analyzed. The young group was associated with higher pathological TNM stage, poorly differentiated tumors, and higher rate of positive distal resection margin (*P* = .010; *P* = .019; *P* = .023 respectively). Young patients were associated with poorer 5-year disease-free survival and local recurrence rates (*P* = .023, *P* = .003 respectively). Cox regression analysis demonstrated that age ≤50 years (Hazard ratio = 2.994, *P* = .038) and higher pathological TNM stage (Hazard ratio = 3.261, *P* = .005) were significantly associated with increased risk for local recurrence. Least absolute shrinkage and selection operator analysis and the time-indepent receiver operating characteristic curve analysis demonstrated that including the age group were superior than that without age group.

Young patients were associated with poorer disease free survival (DFS) and a higher risk for local recurrence in LARC following NCRT. The predicting model basing based on the age group had a better predictive ability. More intense adjuvant treatment could be considered to improve DFS and local control for young patients with LARC following NCRT.

## 1 Introduction

Colorectal cancer (CRC) is the third most common cancer and the second leading cause of cancer-related mortality in the USA.^[1]^ Generally, CRC is thought to be a malignancy affecting mostly on elderly persons. Noteworthy, the incidence of CRC has been on the rise in those under the age of 50 over the last 2 decades. Different from increased risk of comorbidity in old patients, young patients are more likely to present with advanced disease. Nevertheless, most studies focus on the impact of old age on CRC patients, especially over the age of 70.^[2,3]^ Few studies pay special attention to the impact of young age (≤50 years of age) on CRC patients. Given the increasing prevalence in CRC patients age ≤50 years, there is a genuine need to better understand CRC in young patients.

Rectal cancer is different from colon cancer due to biological and clinical hallmarks, as well as embryological origin, anatomy, treatment regimen and thus survival outcome. Neoadjuvant chemoradiotherapy (NCRT) followed by total mesorectal excision (TME) has become the standard of care for locally advanced rectal cancer (LARC). This strategy offers a higher probability of tumor downsizing and downstaging, tumor resectability and sphincter preservation, and better local tumor control.^[4–6]^ Many efforts have been made to evaluate the impact of age on the efficacy of NCRT in patients with rectal cancer, whereas most studies focus on old patients owing to the increased risk of comorbidity and less compliance to neoadjuvant therapy.^[7,8]^ However, few studies pay attention to young LARC patients. Given the aggressive tumor biology of young patients, we hypothesize that the young age might affect the efficacy of NCRT, and thus the survival outcome. Unfortunately, there is no available literature on such issues.

To address the gap in the literature, the present study was aimed to compare the efficacy of NCRT between young (≤50 years of age) and old (>50 years of age) patients with LARC in term of tumor response and survival outcome. Additionally, we further investigated the prognostic significance of age by identifying risk factors for local recurrence in LARC patients following NCRT.

## 2 Patients and method

### 2.1 Availability of data and materials

The data generated or analysed during this study are available from the corresponding author upon reasonable request.

### 2.2 Patient eligibility

This study was a retrospective study. A total of 572 LARC patients who underwent NCRT and radical resection between 2011 and 2015 were identified from our prospectively maintained database. Patient inclusion criteria were as follows:

1) clinical stage II or III (cT3/4 or cN1/2) disease;
2) pathologically proven rectal adenocarcinomas; and
3) tumors located within 12 cm from the anal verge.

Exclusion criteria included:

(1) concurrent with previous or concurrent malignancies;
(2) patients who underwent emergent surgery, palliative resection, or local excision.

This study was approved by the Institutional Review Board (IRB) of Fujian Medical University Union Hospital (2013051).

### 2.3 Treatment protocol

Patient assessments were performed at baseline for tumor staging by means of a digital rectal examination, colonoscopy, chest radiography, abdominopelvic magnetic resonance imaging (MRI) and/or transrectal ultrasound (ERUS). Preoperative long-course radiotherapy consisted of a total dose of 45 Gy to the pelvis, delivered in 25 fractions for 5 consecutive weeks (180 cGy per fraction, 5 days a week), followed by a boost of 5.4 Gy to the primary tumor. Preoperative chemotherapy was initiated on the first day of radiotherapy and included 2 different regimens: 5FU plus oxaliplatin (FOLFOX) or capecitabine plus oxaliplatin (CapeOX).

The operation was performed 6 to 8 weeks after the completion of the radiation. Surgical techniques for rectal cancer, such as TME and high ligation of the inferior mesenteric artery, were routinely performed at our institution. Surgical procedure consisted of low anterior resection (LAR), abdominoperineal resection (APR), or Hartmann's procedure. About 3 to 4 weeks after surgery, patients received postoperative adjuvant chemotherapy (using FOLFOX or CapeOX) for 6 months.

### 2.4 Follow-up

Follow-up protocol was performed every 3 months for the first 3 years, then every 6 months for the next 2 years, and annually thereafter. Physical examination (including digital rectal examination), serum carcinoembryonic antigen (CEA) level, chest X-ray or CT scan, and abdominopelvic MRI or CT scan were performed at each visit. A colonoscopy was performed annually after surgery. Positron emission tomography (PET) was performed when needed. Patient follow-up lasted until death or the cut-off date of December 31, 2018.

### 2.5 Definitions

**Figure 1:**
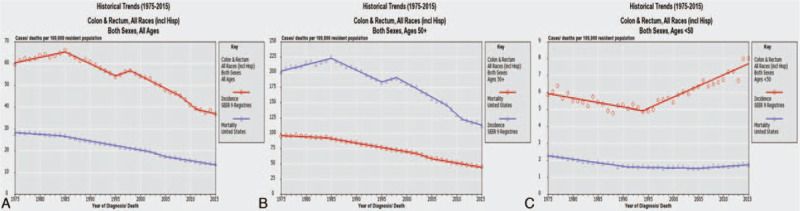
Time trends of CRC incidence and mortality for the population all patients (A), age over 50 (B) and age under 50 (C) a steady decrease in both incidence and mortality in the older age group but an increase in incidence in those younger than age 50. Note the difference in the vertical axis. Graphs generated from Cancer Control Planet https://statecancerprofiles.cancer.gov/historicaltrend/index.php.

Age group was classified according to the Cancer Control Planet classification (https://statecancerprofiles.cancer.gov/historicaltrend/index.php). Time trends in CRC incidence and mortality for all patients, age over and under 50 were shown in Figure 1. Tumor distance from the anal verge was assessed by digital rectal examination, preoperative MRI assessment and intraoperative findings during the operation and pathologic examination. Tumor response to NCRT was graded according to the Rectal Cancer Regression Grade (RCRG) method^[9]^; that is, RCRG 1, sterilization or only microscopic foci of adenocarcinoma remaining, with marked fibrosis; RCRG 2, marked fibrosis but macroscopic disease present; RCRG 3, little or no fibrosis, with abundant macroscopic disease. Pathological complete response (pCR) was defined as the absence of viable tumor cells in the resected specimen, either at the primary site or in the lymph nodes. Postoperative morbidity was classified according to the Clavien-Dindo classification, grades I-II was considered as minor complications, and grades III-V as major complications. Perioperative mortality was defined as any death either within 30 days of surgery or occurring in the hospital.

### 2.6 Statistical analysis

Statistical analysis was performed using SPSS version 20.0 (SPSS INC., Chicago) and R software packages, version 3.5.1 (The R foundation for Statistical Computing (http://www.-rproject.org/) in the study. Categorical variables were presented in frequencies and percentages and were assessed using Chi-square or Fisher exact test. Continuous variables were reported in means and standard deviation between age groups and assessed via the analysis of variance test. Survival outcomes were assessed using the Kaplan-Meier method and log-rank test. A Cox proportional hazards model was performed to identify risk factors for local recurrence. Least absolute shrinkage and selection operator (LASSO) Cox regression model was applied to determine the ideal coefficient for each prognostic feature and estimate the likelihood deviance.^[10–13]^ The coefficients and partial likelihood deviance were calculated with “glmnet” package in R. All variables were entered into LASSO Cox regression model to identify predictors of overall survival. Based on the LASSO Cox regression model analysis, a nomogram was developed by using the R project. The performance of the nomogram was evaluated by time-dependent receiver operating characteristic (ROC) curves. Statistical significance was defined as *P* < .05.

## 3 Result

### 3.1 Patient characteristics

A total of 572 LARC patients were enrolled in our analysis. Among them, 164 (28.7%) were classified into the young group and 408 (81.3%) patients in the old group. The median age in the 2 group was 41.76 and 61.46 years, respectively. Additionally, the American Society of Anaesthesiology grade and post-NCRT CA19-9 level were found significantly different in 2 groups (*P* < .05). No statistical differences were observed between 2 groups in terms of gender, interval time between NCRT and surgery, distance from the anal verge, clinical T stage, clinical N stage, and post-NCRT CEA level, as shown in Table 1.

**Table 1:**
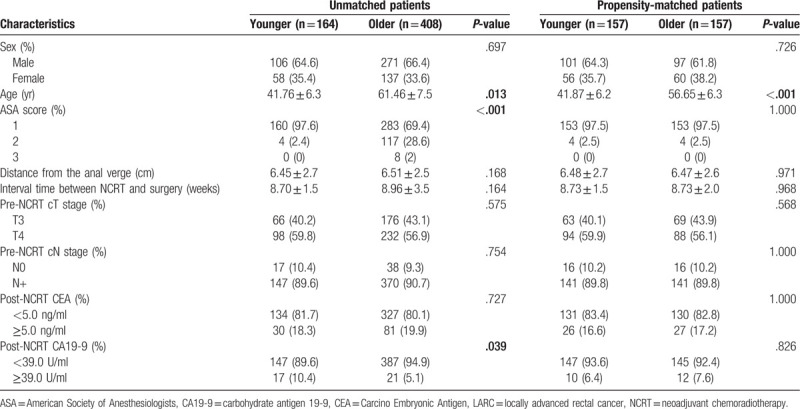
Patient characteristic in patients with LARC following NCRT.

### 3.2 Perioperative outcomes

No significant differences were observed between the groups in terms of estimated blood loss, surgical approach, and preserve organ procedure (Table 2). Compared to the young group, the operation time was significantly decreased in the old group (219.60 ± 50.99 min vs 225.76 ± 60.84 min, *P* = .019) and so as the peri-NCRT complication rates (36.8% vs. 50.0%, *P* = .003). The major complication rates were similar between 2 groups. With regard to the postoperative complication, no significant differences were found between 2 groups in terms of postoperative hospital stay and postoperative complication (*P* = .590, *P* = .705). Similarly, chemotherapy regimen did not differ between the groups (*P* = .229). No re-operation and perioperative mortality were observed between the 2 groups.

**Table 2:**
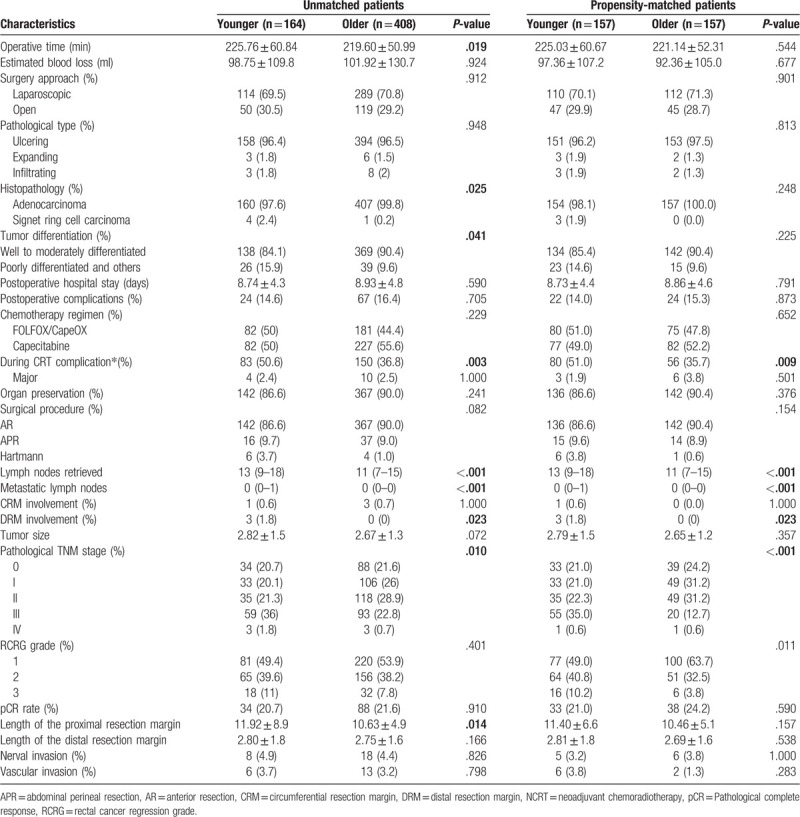
Operative and postoperative outcomes in patients with LARC following NCRT.

### 3.3 Pathological outcomes

Compared to the young group, the old group was associated with less advanced pathological TNM stage (*P* = .010). Moreover, the old group displayed a better result in the histopathology and poorly differentiated (14.6% vs 7.8%, p = 0.019; 14.6% vs 9.3%, *P* = .074). RCRG, pathological type, and pCR rates were similar in both groups (*P* = .401, *P* = .948, *P* = .901). Similarly, neural invasion and vascular invasion did not differ between the groups (*P* = 1.000, *P* = .820). Positive circumferential resection margin was observed in 1 patient (0.6%) in the young group, 3 patients (0.7%) in the old group, and the difference was not significant (*P* = 1.000). Positive distal resection margin rate was significantly higher in the young group (1.8% vs 0, *P* = .023). Additionally, the tumor size and length of the distal resection margin was not significantly different in 2 groups (*P* = .072, *P* = .166), but comparing to the old group, the young group increase the length of the proximal resection margin (11.92 ± 8.9 vs 10.63 ± 4.9, *P* = .014).

### 3.4 Survival outcomes

**Figure 2:**
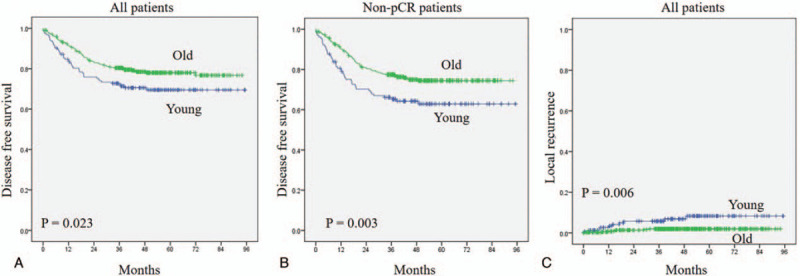
(A) Disease-free survival in all patients, (B) Disease-free survival in Non-pCR patients, (C) cumulative local recurrence between young and old groups. pCR = pathological complete response.

After a mean follow-up of 48 months (range 3–95 months), the 5-year disease-free survival significantly decreased in the young group compared with the old group (young 72.7%, old 78.0%, *P* = .023), as shown in Figure 2A. In subgroup analysis, we compared the 5-year disease-free survival between the pCR and non-pCR group. The result demonstrated that there was no statistical difference in the pCR rate between the 2 groups. However, the young group was associated with worse survival in the non-pCR than the old group (*P* = .006), as shown in Figure 2B. The 5-year cumulative local recurrence rate was 8% in the young group, slightly higher than 2% in the old group (*P* = .003), as shown in Figure 2C.

### 3.5 Prognostic factors for local recurrence and overall survival

On univariate analysis, age (Hazard ratio [HR] = 3.846, *P* = .006), tumor size (HR = 1.602, *P* = .001), higher pathological TNM stage (HR = 4.433, *P* < .001), RCRG grade (HR = 2.552, *P* = .005), post-NCRT CEA level (HR = 3.725, *P* = .008), post-NCRT CA19-9 level (HR = 3.958, *P* = .031), vascular invasion (HR = 0.200, *P* = .033), and DRM involvement (HR = 0.022, *P* < .001) were independently associated with local recurrence in LARC patients following NCRT. Cox regression analysis demonstrated that ≤50 years of age (HR = 2.994, *P* = .038) and higher pathological TNM stage (HR = 3.261, *P* = .005) remained significantly associated with increased risk of local recurrence, as demonstrated in Table 3.

**Table 3:**
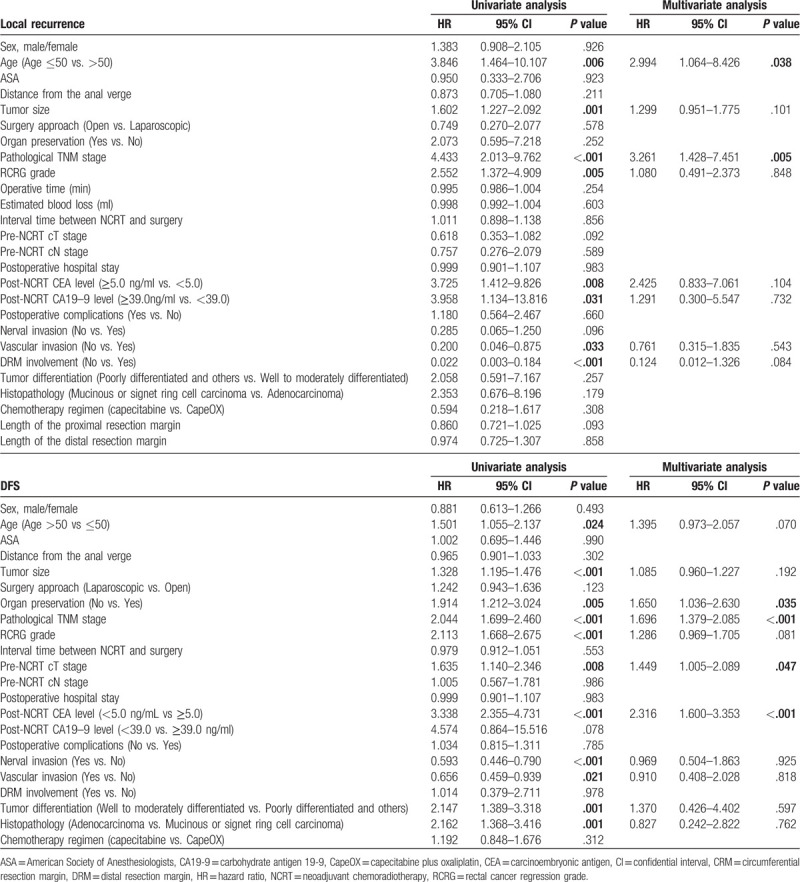
Univariate and multivariate analysis of predictive factors for local recurrence and DFS in patients with LARC following NCRT (n = 572).

**Figure 3:**
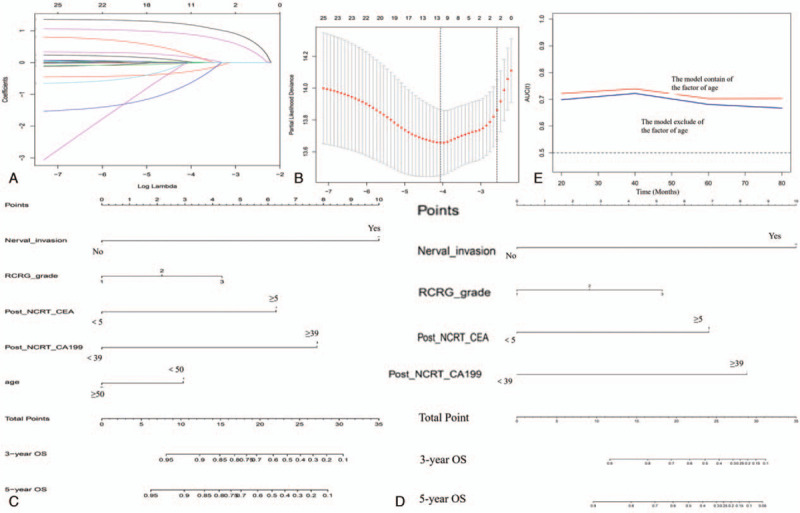
Construction of the factors for the overall survival. (A) LASSO coeffi cient profi les of the 29 factors, (B) The AUC was estimated with cross-validation technique and the largest lambda value was chosen when the cross-validation error was within 1 standard error of the minimum. (C) and (D) Nomogram developed for prediction of overall survival, (C) the model with age group and (D) the model without age group. (E) Time-dependent AUC curves of 2 models for the prediction of overall survival. AUC = area under the curves.

LASSO analysis was used to explore significant predictors for overall survival. The result demonstrated that RCRG grade, post-NCRT CEA level, post-NCRT CA19-9 level, nerval invasion, and age group were the top-5 significant factors (Fig. 3A and B). By incorporating the significant determinants in the LASSO analysis, 2 predicting nomograms for overall survival in LARC patients after NCRT were developed with/without age group, as demonstrated in Figure 3C and D. A comparison of the time-dependent area under the curves (AUC) of ROC curves of nomograms for the prediction of OS showed that the AUCs for all of them were relatively stabilized after surgery, but that the AUC of the model containing the age group tended to be higher than the other model without age group at all times tested (Figure 3E).

### 3.6 Subgroup analysis

**Figure 4:**
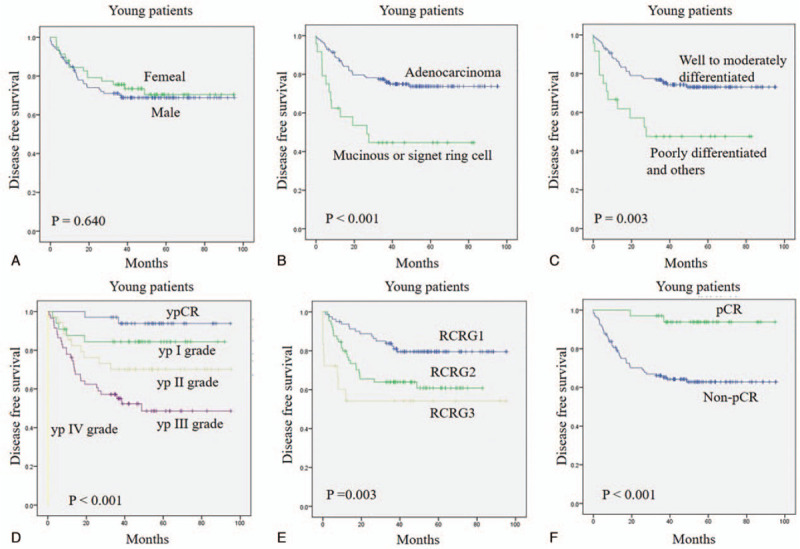
Disease-free survival in young patients between (A) gender group, (B) histopathology group, (C) tumor differentiation group, (D) pathological TNM stage group, (E) RCRG grade group, (F) pCR group. pCR = pathological complete response, RCRG = rectal cancer regression grade.

Having shown the prognostic significance of young patients in local recurrence, we further explored the prognostic factors for disease free survival (DFS) in young patients. The results demonstrated that poorly differential, mucinous adenocarcinoma, higher pathological TNM stage, worse RCRG grade, and non-pCR patients were associated with a worse DFS rate (all *P* < .05, Figure 4B-F). Not surprisingly, gender was not correlated with DFS in young patients (*P* > .05), as shown in Figure 4A.

## 4 Discussion

Age is an important factor affecting the efficiency and toxicity of NCRT in patients with rectal cancer. To our best knowledge, few studies focused on young LARC patients following NCRT. In the present study, we explored the efficiency and toxicity of NCRT between young (≤50 years of age) and old (>50 years of age) patients with LARC. The result demonstrated that LARC patients under the age of 50 had a worse 5-year DFS compared with those over the age of 50, without affecting postoperative complications.

As reported by the National Institutes of Health, the incidence and mortality of CRC patients under the age of 50 are relatively higher than those over the age of 50 in recent decades. Several studies have reported that young LARC patients presented poor pathological features and advanced stage compared with older patients.^[14–16]^ Additionally, poor pathological features could also prompt a worse tumor response to NCRT.^[17–23]^ However, response to NCRT in the young LARC patients has not yet been clarified. Herein, we demonstrated that young LARC patients displayed poorer pathological features, such as a higher probability of mucinous or signet ring cell and poorly differentiated tumors, which was in accordance with previous studies.^[18,19,21]^ These results indicated the young-onset LARC patients had poorly pathological features than the old group; however, the 2 groups had similar tumor response to NCRT. Unfortunately, the mechanism responsible for this phenomenon is not yet available.

Additionally, few studies have explored survival outcomes in young LARC patients receiving TME followed NCRT; and survival outcome between young and old LARC patients is still controversial. Some studies have suggested a relatively worse prognosis in young CRC patients because of delayed diagnosis and more advanced tumor stage. Other studies have reported a comparable survival between young and old patients after adjustment for comorbidity-related variables. In this study, we explored the DFS in the 2 groups; the result demonstrated that young LARC patients had a worse 5-year DFS compared with old patients. Interestingly, we revealed that young LARC patients had a similar pCR and RCRG1 rate, but higher recurrence rates compared with the old patients. To further explore the impart of young LARC age on OS of LARC patient following TME and NCRT, we constructed a predictive model based on the nomogram. In addition, LASSO was performed to select appropriate factors enrolled in the nomogram to avoid overfitting in the model.^[10–13]^ The results of time-independent ROC demonstrated that age played an important role in the model, and the AUC of the model including age group was higher than the model without age group. Together, these findings suggested that the young age was associated with a poorer DFS and OS, and proven to be a significant determinant affecting the survival of LARC patients. PCR has been used as a surrogate endpoint for early efficacy and long-term survival in LARC following NCRT. In the present study, we found that the response to NCRT in 2 groups was comparable, but the old group had a better survival outcome. Therefore, we further explored whether age was a factor affecting DFS in different tumor response (pCR versus non-pCR) groups. Subgroup analysis demonstrated that the recurrence rates are no statistically different between young and old LARC patients achieving pCR. Interestingly, in the non-pCR subgroup, the DFS rate was lower in young patients with LARC. These results suggested the prognostic value of age in non-pCR LARC patients. The possible explanation might be that pCR is an important endpoint surrogate which could improve survival outcomes in LARC patients following NCRT.

NCRT is the standard of care for LARC patients which could improve local but not distant tumor control.^[24,25]^ Our results demonstrated that the young group (≤50 years of age) and a higher pathological TNM stage were independent risk factors for local recurrence. Owing to a lack of high-level evidence, there is currently no uniform consensus regarding the impact of age on the efficacy of NCRT and outcome of LARC patients. Nevertheless, in the other tumors receiving CRT, age has been proven to be an important factor for survival benefits and risk of complication. Age-related decisions about the use of NCRT in patients with rectal cancer are a difficult and complex process, considering the balance between the likely risks and survival benefits. In our study, young patients had worse survival compared with the old patients, especially in the non-pCR group. Thus, more intense adjuvant chemotherapy could be considered in young LARC patients in order to improve the survival outcome. Having shown the prognostic value of young age in local recurrence, we further demonstrated that poorly differential, mucinous adenocarcinoma, higher pathological TNM stage, worse RCRG grade, and non-pCR patients were associated with a worse DFS rate in young patients. These findings suggested that individualized surveillance and intensified adjuvant therapy could be considered for such patients.

There are several limitations that warrant discussion. First, the present study was subjected to potential selection bias due to the retrospective design. Second, age-related comorbidities, such as the Charlson score, was not evaluated in the present study due to the lack of adequate data. Third, the impact of gene profiling was not assessed owing to the lack of complete medical records in some cases. Given these limitations, we believe this study adds to the understanding of the impact of young age on the efficacy of NCRT and oncological outcomes in patients with LARC following NCRT.

In this cohort study of 572 LARC patients treated at a single high-volume cancer center, young LARC patients (≤50 years of age) was associated with poorer DFS and a higher risk for local recurrence. In addition, age was identified as a significant prognostic determinant for OS in the predicting model. More intense adjuvant treatment could be considered to improve the DFS and local control for young patients with LARC following NCRT. Larger-scale prospective clinical trials are warranted to confirm the above findings.

## Acknowledgment

The authors thank all the staff in Department of Colorectal surgery, Fujian Medical University Union Hospital, Fuzhou, Fujian Province, People's Republic of China.

## Author contributions

YYZ and GXG participated in all experimental work and drafted the paper. XL and YW collected the data. BC and SFL analyzed the data. All the authors have read and approved the final manuscript. All authors contributed toward data analysis, drafting and revising the paper and agree to be accountable for all aspects of the work.

**Conceptualization:** Yiyi Zhang, Yibin Su.

**Data curation:** Yiyi Zhang.

**Methodology:** Yiyi Zhang.

**Resources:** Yiyi Zhang.

**Software:** Yiyi Zhang.

**Validation:** Yibin Su.

**Visualization:** Yibin Su.

**Writing – original draft:** Yiyi Zhang.

**Writing – review & editing:** Yiyi Zhang, Yibin Su.
